# Glucotropaeolin Promotes Apoptosis by Calcium Dysregulation and Attenuates Cell Migration with FOXM1 Suppression in Pancreatic Cancer Cells

**DOI:** 10.3390/antiox12020257

**Published:** 2023-01-23

**Authors:** Woonghee Lee, Gwonhwa Song, Hyocheol Bae

**Affiliations:** 1 Institute of Animal Molecular Biotechnology, Department of Biotechnology, College of Life Sciences and Biotechnology, Korea University, Seoul 02841, Republic of Korea; 2 Department of Oriental Medicinal Biotechnology, College of Life Sciences, Kyung Hee University, Yongin 17104, Republic of Korea

**Keywords:** pancreatic cancer, glucotropaeolin, migration, calcium dysregulation, apoptosis, FOXM1

## Abstract

Pancreatic ductal adenocarcinoma (PDAC) has naturally aggressive characteristics including postoperative recurrence, resistance to conventional treatment, and metastasis. Surgical resection with chemotherapeutic agents has been conducted as the major treatment for PDAC. However, surgical treatment is ineffective in the case of advanced cancer, and conventional adjuvant chemotherapy, including gemcitabine and 5-fluorouracil, show low effectiveness due to the high drug resistance of PDAC to this type of treatment. Therefore, the development of innovative therapeutic drugs is crucial to solving the present limitation of conventional drugs. Glucotropaeolin (GT) is a glucosinolate that can be isolated from the Brassicaceae family. GT has exhibited a growth-inhibitory effect against liver and colon cancer cells; however, there is no study regarding the anticancer effect of GT on PDAC. In our study, we determined the antiproliferative effect of GT in PANC-1 and MIA PaCa-2, representative of PDAC. We revealed the intracellular mechanisms underlying the anticancer effect of GT with respect to cell viability, reactive oxygen species (ROS) accumulation, alteration of mitochondrial membrane potential (MMP), calcium dysregulation, cell migration, and the induction of apoptosis. Moreover, GT regulated the signaling pathways related to anticancer in PDAC cells. Finally, the silencing of the forkhead box protein M, a key factor regulating PDAC progression, contributes to the anticancer property of GT in terms of the induction of apoptosis and cell migration. Therefore, GT may be a potential therapeutic drug against PDAC.

## 1. Introduction

Pancreatic cancer has been considered a devastating disease in the world with the third leading cause of estimated deaths and the lowest survival rate (11%) among men and women combined in the United States [1]. Due to the absence of an effective standard program for screening patients at early stages, most patients with pancreatic cancer have reached an advanced stage by the time of diagnosis [2]. Pancreatic ductal adenocarcinoma (PDAC), accounting for above 90% of entire patients with pancreatic cancer, has naturally aggressive characteristics including postoperative recurrence, resistance to conventional treatment, and metastasis [3,4]. Surgical resection with chemotherapeutic agents has been conducted as the major treatment for PDAC. However, surgical treatment is ineffective in the case of advanced cancer, and conventional adjuvant chemotherapy, including gemcitabine and 5-fluorouracil, shows low effectiveness due to the high drug resistance of PDAC to this type of treatment [5]. Moreover, although new therapeutic strategies including FOLFIRINOX and the multidrug regimens of gemcitabine, nab-paclitaxel, cisplatin, and capecitabine have been proposed to solve the chemoresistance to PDAC, they still exhibited low efficacy in PDAC patients at advanced stages [6,7]. Therefore, the development of innovative therapeutic drugs is crucial to solving the present limitation of conventional drugs.

Glucotropaeolin (GT) is a glucosinolate that can be isolated from the Brassicaceae family, such as cabbage, broccoli, and garden cress [8]. Benzyl isothiocyanate (BITC), which is enzymatically hydrolyzed from GT, has various pharmacological and biomedical activities, including anti-inflammatory, antioxidative, and antiangiogenic activities [9,10,11]. Additionally, numerous studies have reported that BITC has anticancer effects in diverse cancers, including pancreatic, bladder, breast, and lung cancer [12,13,14,15]. A recent study has revealed that GT, the precursor of BITC, also exhibited a growth-inhibitory effect against hepatocellular carcinoma and colon adenocarcinoma cell lines [16,17]. However, there is no study regarding the anticancer effect of GT on PDAC.

Forkhead box M1 (FOXM1), representative of the forkhead box transcription factors, is essential for modulating cell proliferation and cell cycle transition [18]. Evidence has recently suggested that FOXM1 is overexpressed in PDAC and that its overexpression is closely involved in the invasion, metastasis, and poor prognosis of PDAC [19,20,21]. Moreover, FOXM1 also enhances the expression of epithelial–mesenchymal transition (EMT)- markers and resistance to apoptosis [22,23]. Therefore, the downregulation of FOXM1 could be a promising anticancer strategy for preventing PDAC invasion and metastasis.

In our study, we determined the antiproliferative effect of GT in PANC-1 and MIA PaCa-2, representative of PDAC. This study tried to (1) reveal molecular alterations caused by the anticancer effect of GT with respect to cell viability, reactive oxygen species (ROS) accumulation, the induction of apoptosis, alteration of mitochondrial membrane potential (MMP), calcium dysregulation, and cell invasiveness; (2) identify the signaling pathways involved in an anticancer activity regulated by GT in PDAC cells; and (3) investigate the contribution of FOXM1 in the induction of apoptosis through disrupting calcium homeostasis and cell migration in PDAC cells.

## 2. Materials and Methods

### 2.1. Cell Culture

The human PDAC cell lines, MIA PaCa-2 and PANC-1 were purchased from the Korean Cell Line Bank (Seoul, Republic of Korea). They were raised in the presence of DMEM (Hyclone, Carlsbad, CA, USA) supplemented with 1% penicillin–streptomycin (Hyclone) and 10% inactivated fetal bovine serum (FBS, Hyclone). For the following experiments, the cells were seeded into 60 mm dishes (3 × 10^5^ cells/well), 6-well (2 × 10^5^ cells/well), or 96-well plates (2 × 10^4^ cells/well), and they were maintained until 70~80% confluence. Then, the cells were incubated in a serum-free medium for 24 h and treated with various concentrations of GT.

### 2.2. Chemicals

Glucotropaeolin (GT, Cat: PHL89216) was obtained from Sigma-Aldrich (St. Louis, MO, USA) and dissolved in dimethyl sulfoxide (DMSO). Table 1 shows detailed information about the antibodies used in this study.

### 2.3. Spheroids Analysis

The spheroids formation analysis for the PDAC cell lines was performed using the hanging drop method. Briefly, the PDAC cells (10,000 cells/drop) were hung onto the inside of a petri dish cover with or without 100 µM of GT for 3 days. The morphology of each spheroid was observed using a Leica DM3000 microscope and then the captured images were moved to ImageJ 1.53e (NIH, Bethesda, MD, USA). The number of PDAC cell colonies and the relative total area were quantified using ImageJ.

### 2.4. Cell Viability

We assessed the viability of PDAC cells in response to GT with Cell Proliferation Kit I (Roche, Basel, Switzerland). In brief, PDAC cells were incubated with various doses of GT for 48 h, and then MTT labeling solution was added to the cells. After incubating at 37 °C for 4 h, a solubilization buffer was added to each cell. The optical density of purple formazan crystals was evaluated with a microplate reader (Molecular Devices Filter Max F5; San Francisco, CA, USA) at 595 nm and 620 nm.

### 2.5. Immunoblotting Analysis

The protein was obtained from PDAC cells followed by immunoblotting, as described in our previous study [24].

### 2.6. Reactive Oxygen Species (ROS) Production Analysis

The PDAC cells seeded onto 6-well plates (2 × 10^5^ cells/well) were treated with GT (0, 20, 50, and 100 µM) in the presence of DMEM containing 2% FBS for 1 h. Then, the cells were stained using DCFH-DA (Sigma-Aldrich) for 30 min and rinsed with PBS. Fluorescent levels were evaluated with a flow cytometer (BD Accuri C6, BD Biosciences, San Jose, CA, USA) and BD Accuri C6 software.

### 2.7. Analysis of Mitochondrial Membrane Potential

The depolarization of the mitochondrial membrane was assessed using a mitochondrial staining kit (Cat: CS0390, Sigma-Aldrich). Briefly, the PDAC cells were incubated with GT (0, 20, 50, and 100 μM) for 48 h. Then, the PDAC cells were detached with 0.25% trypsin–ethylenediaminetetraacetic acid (EDTA) and harvested. After being stained with JC-1 dye for 20 min, the PDAC cells were rinsed with 1X JC-1 buffer. The alteration of MMP was evaluated using a flow cytometer (BD Accuri C6, BD Biosciences) and BD Accuri C6 software.

### 2.8. Annexin V and Propidium Iodide (PI) Staining

The induction of apoptosis in PDAC cells by GT (0, 20, 50, and 100 µM) and the attenuation of FOXM1 with or without 100 µM of GT were assessed using apoptosis detection kit I (BD Biosciences, Franklin Lakes, NJ, USA), as described in a previous study [24].

### 2.9. Mitochondrial and Intracellular Ca^2+^ Analysis

The mitochondrial and intracellular calcium concentrations in PDAC cells were measured using fluorescent dye Rhod-2 (Invitrogen, Carlsbad, CA, USA) and Fluo-4 (Invitrogen), respectively. Briefly, the PDAC cells were treated with GT with or without 1,2-bis-(2-aminophenoxy)ethane-N,N,N′,N′-tetraacetic acid tetra(acetoxymethyl) ester (BAPTA-AM) or 2-aminoethoxydiphenyl borate (2-APB) for 48 h. After being stained with Rhod-2 at 4 °C for 30 min and Fluo-4 at 37 °C for 20 min, cells were rinsed, and the emission intensities of the fluorescent dyes were detected using a flow cytometer (BD Accuri C6, BD Biosciences) and BD Accuri C6 software.

### 2.10. Quantitative PCR (qPCR)

Total RNA was isolated using TransZol Up reagent (TransGen Biotech, Beijing) according to the manufacturer. Complementary DNA was synthesized using AccuPower^®^ RT PreMix (Bioneer, Daejeon, Republic of Korea), and products of interest were amplified by qPCR using SYBR green and the CFX Connect Real-Time System (Bio-Rad Laboratories Inc., Hercules, CA, USA), as described in the previous study [24]. Table 2 shows the specific primers used in this study.

### 2.11. Migration Assay

The migratory ability of PDAC cells seeded onto SPL Hanging membranes (SPL Life Sciences, Pocheon, South Korea) in the presence of GT (100 μM) was analyzed, as described in the previous study [24].

### 2.12. Invasion Assay

The invasiveness of PDAC cells seeded onto 35 mm culture dishes (ibidi, Munich, Germany) was analyzed, as described in the previous study [24].

### 2.13. Small Interference RNA (siRNA) Transfection

The attenuation of FOXM1 expression was conducted by transfection of siFOXM1 with Lipofectamine 2000 (Invitrogen) in accordance with the manufacturer’s instructions (Bioneer, Daejeon, Republic of Korea). The sequence of siFOXM1 was 5′-AGU-UUC-CAG-CUG-GGA-UCA-ATT-3′ for the sense strand and 5′-UUG-AUC-CCA-GCU-GGA-AAC-UTT-3′ for the antisense strand. siFOXM1 (final concentration of 50 nM) and nontargeting control siRNA (siControl; final concentration of 20 nM) were obtained from Bioneer based on the genome-wide predesigned siRNA library. Briefly, siRNA and lipofectamine were dissolved in Opti-MEM (Gibco-BRL Life Technologies, Waltham, MA, USA), respectively, and incubated for 5 min at room temperature. Then, both mixtures were blended and incubated for another 20 min. The PDAC cells were treated for 24 h, then rinsed with PBS, followed by the treatment with or without GT.

### 2.14. Statistics

All data were calculated with unpaired *t*-tests between two groups or analysis of variance (ANOVA), followed by Tukey’s post hoc test among various groups using GraphPad Prism 7. All data were obtained in triplicates. *p*-values below 0.05 were regarded as statistically significant. Data are presented as means ± standard deviation.

## 3. Results

### 3.1. GT Suppresses Cell Proliferation and Elevates ROS Production in PDAC Cells

**Figure 1:**
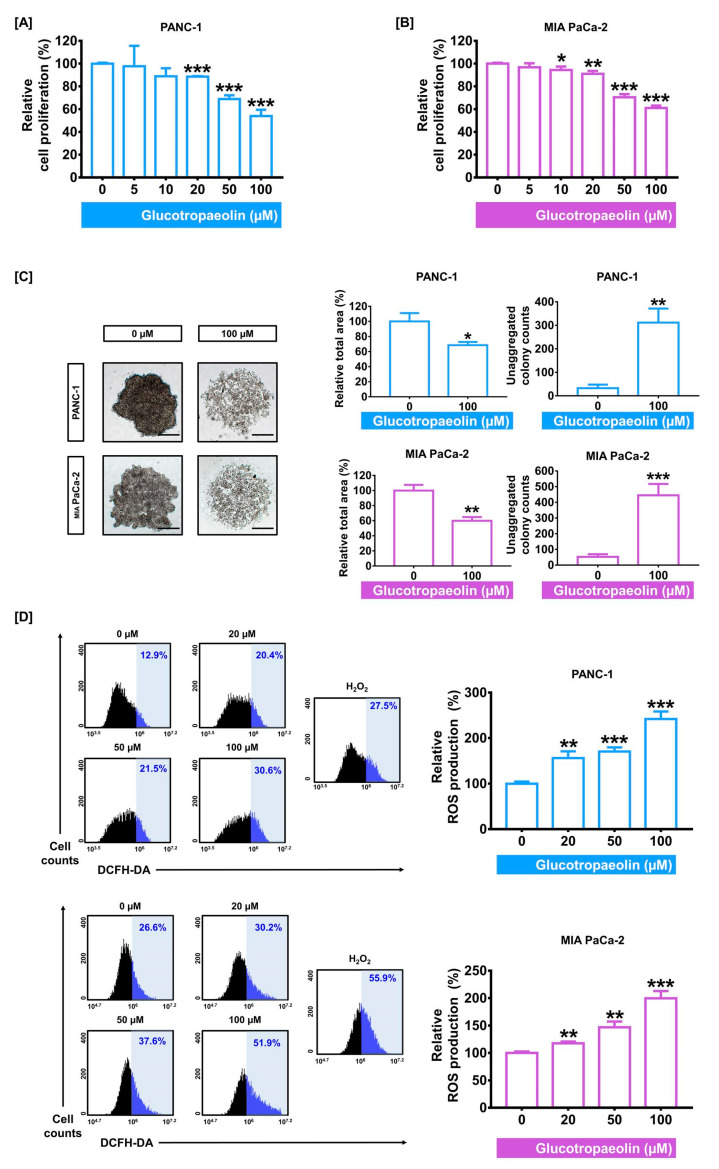
The effect of glucotropaeolin (GT) on proliferation and reactive oxygen species (ROS) accumulation of human pancreatic ductal adenocarcinoma (PDAC) cells. (**A**,**B**) The PDAC cells were treated with various doses of GT for 48 h. In response to GT, an investigation of cell viability was performed in both PDAC cells. Above 20 µM of GT, relative viability was gradually reduced. (**C**) The morphology of the PDAC cell spheroid with or without GT (100 µM). Relative total area and unaggregated colony number were represented by a bar graph. Scale bar: 100 µm. (**D**) ROS accumulation was analyzed by detecting the emission of DCF fluorescence with flow cytometry. H_2_O_2_ (100 µM) was the positive control group. Triplicated experiments were performed. The levels of statistical significance between control and GT-treated groups are indicated by asterisks, as confirmed by unpaired *t*-tests or one-way analysis of variance (ANOVA), followed by Tukey’s post hoc test. * *p* < 0.05, ** *p* < 0.01, and *** *p* < 0.001 indicate GT-induced significance.

To evaluate the inhibitory effect of GT on the proliferation in human PDAC cells (PANC-1 and MIA PaCa-2), both cells were incubated for 48 h in the presence of GT (0, 5, 10, 20, 50, and 100 µM). At the concentration of GT above 20 µM, the proliferation of both PDAC cells was significantly reduced. Based on this result, we set the concentration of GT as above 20 µM in the following experiments. Noteworthily, the inhibitory effect on cell proliferation at 100 µM of GT was 46.1% and 39.0% in PANC-1 and MIA PaCa-2, respectively (*p* < 0.001 in both cells) (Figure 1A,B). Additionally, we confirmed the morphological and physical alterations in the spheroids formation induced by the GT treatment in PDAC cells by using the hanging drop method (Figure 1C). After treatment with 100 µM of GT, the relative total area of each spheroid was reduced by 31.5% (*p* < 0.05) in PANC-1 and 40.0% (*p* < 0.01) in MIA PaCa-2, compared to the control (0 µM). Moreover, GT treatment considerably increased the number of unaggregated cell colonies in both cells from 33 colonies to 312 colonies of PANC-1 (*p* < 0.01) and from 53 colonies to 445 colonies of MIA PaCa-2 (*p* < 0.001).

Next, based on the previous report that the modulation of ROS levels in cancer cells can exert an anticancer effect in cancer therapy [25], we investigated the ROS production caused by GT in PDAC cells. Flow cytometry data indicated that GT treatment gradually elevated ROS production in both PDAC cells (Figure 1D). When compared to the control, after treatment with 100 µM of GT, the relative ROS levels increased by up to 242% in PANC-1 and 200% in MIA PaCa-2 (*p* < 0.001 in both cells). These increments in ROS levels were comparable to those by H_2_O_2_ (100 µM), the positive control. Altogether, these findings suggest that GT exerts an antiproliferative effect and promotes ROS accumulation in PDAC cells. 

### 3.2. GT Provokes the Induction of Apoptosis and Lowers Mitochondrial Membrane Potential (MMP) in PDAC Cells

**Figure 2:**
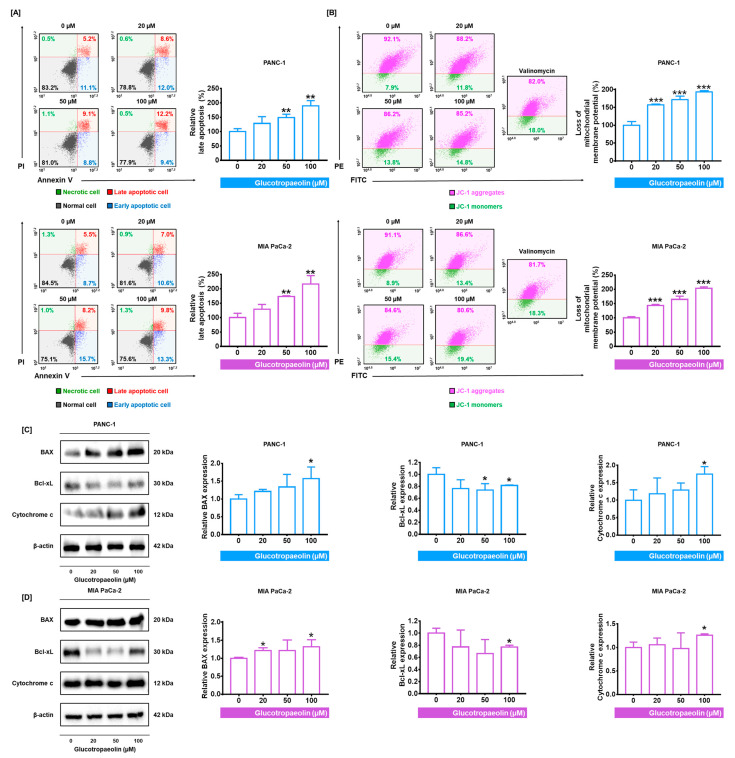
Alteration of apoptotic cell rate and mitochondrial membrane potential (MMP) by glucotropaeolin (GT) in pancreatic ductal adenocarcinoma (PDAC) cells. (**A**) The rate of apoptotic PDAC cells was detected with Annexin V/PI dyes after GT treatment for 48 h. (**B**) Changes in MMP in PDAC cells induced by GT treatment for 48 h were analyzed with JC-1 using flow cytometry. One µg/mL valinomycin was the positive control group. (**C**,**D**) Protein expression of Bcl-xL, BAX, and cytochrome c in PDAC cells after GT treatment for 48 h. Triplicated experiments were performed. The levels of statistical significance between control and GT-treated groups are indicated by asterisks, as confirmed by one-way analysis of variance (ANOVA), followed by Tukey’s post hoc test. * *p* < 0.05, ** *p* < 0.01, and *** *p* < 0.001 represent GT-induced significance.

Based on the results that GT suppresses cell proliferation and stimulates ROS accumulation in PDAC cells, we then investigated whether the GT treatment of PDAC cells can induce apoptosis. Flow cytometry data of Annexin V/PI assay demonstrated that late apoptosis increased in response to dose-dependent GT treatment in both PDAC cells (Figure 2A). Compared to the control group, the treatment of 100 µM GT increased the relative late-apoptotic PANC-1 and MIA PaCa-2 levels by up to 189% (*p* < 0.01) and 216% (*p* < 0.01), respectively. As the process of apoptosis is often accompanied by the depolarization of the mitochondrial membrane [26], we confirmed the alteration of MMP with JC-1 dye in response to GT in PDAC cells. The JC-1 monomers rate, which corresponds to the loss of MMP, was increased by the 100 µM GT by up to 194% in PANC-1, and 203% in MIA PaCa-2 (*p* < 0.001 in both) (Figure 2B). These increments were comparable to those using 1 µg/mL valinomycin, the positive control, in both PDAC cells. Valinomycin can induce rapid apoptosis by reducing MMP [27]. Next, we further confirmed the protein expression related to apoptosis in PDAC cells (Figure 2C,D). In response to 100 µM of GT, compared to the control group, the expression of BAX in PANC-1 and MIA PaCa-2 was increased 1.64-fold and 1.46-fold (both *p* < 0.05), respectively. Additionally, Bcl-xL expression was 0.82-fold in PANC-1 and 0.77-fold in MIA PaCa-2 (both *p* < 0.05) in response to 100 µM of GT. Moreover, cytochrome c expression was also increased by 100 µM of GT (1.74-fold in PANC-1 and 1.26-fold in MIA PaCa-2) (both *p* < 0.05). Collectively, these results indicate that GT promotes the induction of apoptosis and causes a reduction in MMP in PDAC cells.

### 3.3. GT Induces Mitochondrial Dysfunction via Calcium Dysregulation in PDAC Cells

**Figure 3:**
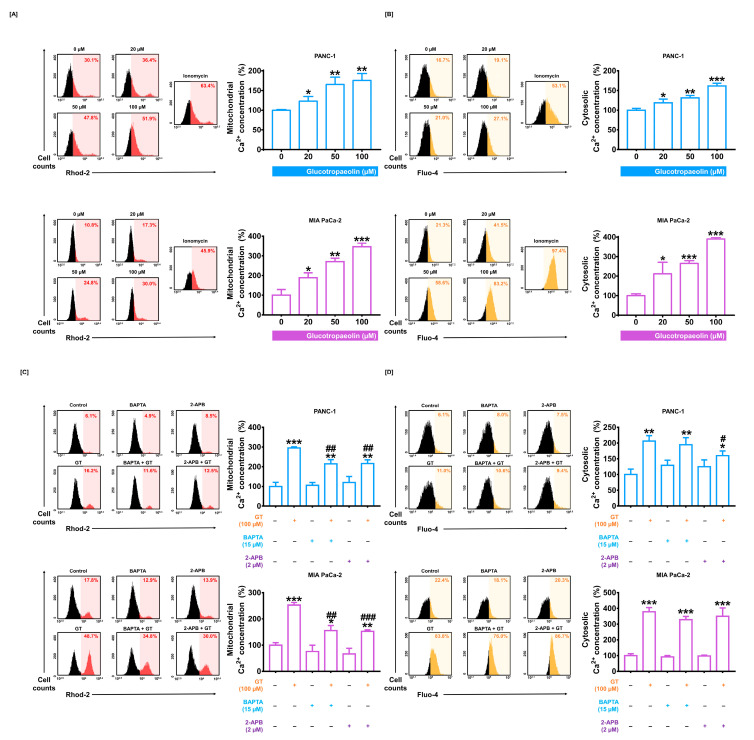
Glucotropaeolin (GT) elevates mitochondrial and cytosolic calcium levels in pancreatic ductal adenocarcinoma (PDAC). (**A**) The change in mitochondrial calcium levels induced by GT treatment for 48 h was evaluated with the Rhod-2 assay using flow cytometry. The treatment with ionomycin (10 µM) was the positive control group. (**B**) Dose-dependent cytosolic calcium level induced by GT treatment for 48 h was evaluated with the Fluo-4 assay using flow cytometry. The treatment with ionomycin (10 µM) was the positive control group. (**C**) Flow cytometry results for the alteration of mitochondrial calcium levels by the combination of GT with BAPTA/AM or 2-APB for 48 h. (**D**) Flow cytometry results for the alteration of cytosolic calcium level by the combination of GT with BAPTA/AM or 2-APB for 48 h. Triplicated experiments were performed. The levels of statistical significance between the control and GT-treated groups are indicated by asterisks (* *p* < 0.05, ** *p* < 0.01, *** *p* < 0.001) and between the GT-only treatment group and the co-treatment group by crosshatches (# *p* < 0.05, ## *p* < 0.01, and ### *p* < 0.001), as confirmed by one-way analysis of variance (ANOVA), followed by Tukey’s post hoc test.

To evaluate the changes in calcium concentration in mitochondria and cytosol, we performed Rhod-2 and Fluo-4 assays with flow cytometry, respectively. Flow cytometry analysis verified that GT (100 µM) increased mitochondrial calcium levels by up to 175.5% in PANC-1 (*p* < 0.01) and 346.0% in MIA PaCa-2 (*p* < 0.001) compared to the control group (Figure 3A). Cytosolic calcium levels also increased using 100 µM of GT treatment in PANC-1 (by up to 161.4%; *p* < 0.001) and in MIA PaCa-2 (by up to 390.3%; *p* < 0.001) (Figure 3B). The increment in mitochondrial calcium level induced by GT was attenuated by calcium chelators: BAPTA and 2-APB (PANC-1: from 295.2% to 214.7% and 216.3%, respectively; both *p* < 0.01, and MIA PaCa-2: from 252.4% to 155.6%; *p* < 0.01 and to 152.7%; *p* < 0.001, respectively) (Figure 3C). Moreover, the co-treatment with calcium chelators significantly prevented increases in cytosolic calcium levels in PANC-1, but nonsignificantly in MIA PaCa-2 (Figure 3D).

### 3.4. Anticancer Signaling Pathways Induced by GT in PDAC Cells

**Figure 4:**
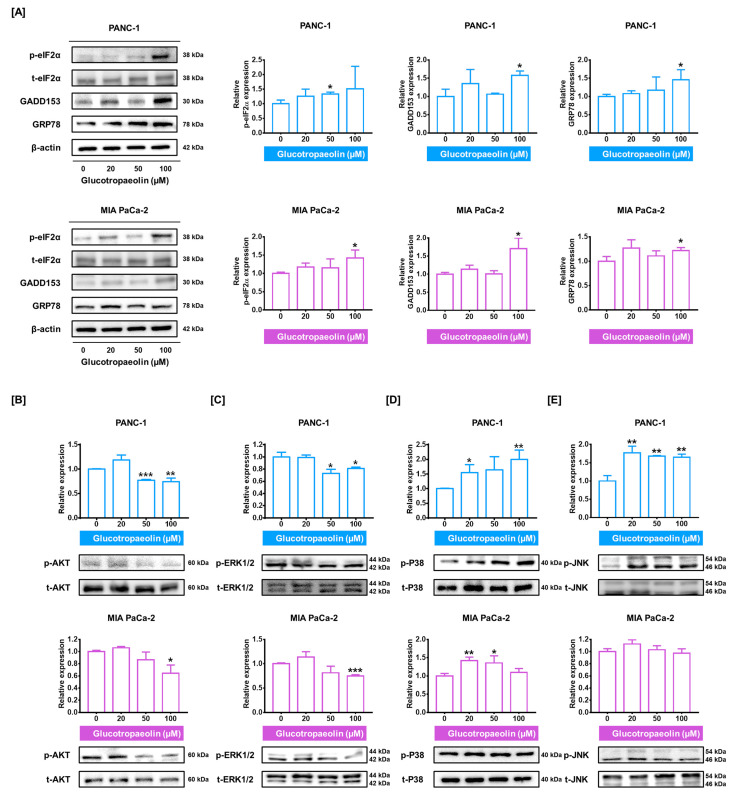
Glucotropaeolin (GT) anticancer signaling pathways in pancreatic ductal adenocarcinoma (PDAC) cells. (**A**) ER stress-related proteins (GRP78, GADD153, and phosphorylated eIF2α) expression indicated by Western blotting. (**B**–**E**) Changes in (**B**) phosphorylated (p)-AKT, (**C**) p-ERK1/2, (**D**) p-P38, and (**E**) p-JNK protein levels in GT-treated PDAC cells. Triplicated experiments were performed. The levels of statistical significance between control and GT-treated groups are indicated by asterisks, as confirmed by one-way analysis of variance (ANOVA), followed by Tukey’s post hoc test (* *p* < 0.05, ** *p* < 0.01, and *** *p* < 0.001).

Then, we investigated the GT-stimulating signaling pathways related to PDAC cell progression. With respect to the calcium dysregulation induced by the GT treatment shown in Figure 3, we investigated the endoplasmic reticulum (ER) stress-regulatory proteins because perturbation of calcium homeostasis can evoke ER stress activation [28]. Immunoblotting results suggested that GT activated ER stress-related proteins including phosphorylated eIF2α, GADD153, and GRP78 in PDAC cells (Figure 4A). Subsequently, the proteins classified as AKT and MAPK signaling pathways were investigated. In response to 100 µM of GT, phosphorylated AKT in PANC-1 and MIA PaCa-2 decreased to 0.75-fold (*p* < 0.01) and 0.64-fold (*p* < 0.05), respectively (Figure 4B). Similarly, the phosphorylation levels of ERK1/2 in both PDAC cells were reduced by the 100 µM GT treatment to 0.81-fold and 0.75-fold (*p* < 0.05 and *p* < 0.001), respectively (Figure 4C). Conversely, the expression of phosphorylated P38 was increased by the 20 µM of GT treatment, by up to 1.55-fold and 1.42-fold (*p* < 0.05 and *p* < 0.01), respectively (Figure 4D). On the other hand, phosphorylated JNK in PANC-1 was significantly increased by 100 µM of GT, by up to 1.65-fold (*p* < 0.01); however, this change was not as significant in MIA PaCa-2 (Figure 4E). Collectively, GT stimulates ER stress-regulatory proteins and regulates the AKT and MAPK signaling pathways in PDAC cells.

### 3.5. GT Weakens Cell Invasiveness in PDAC Cells

**Figure 5:**
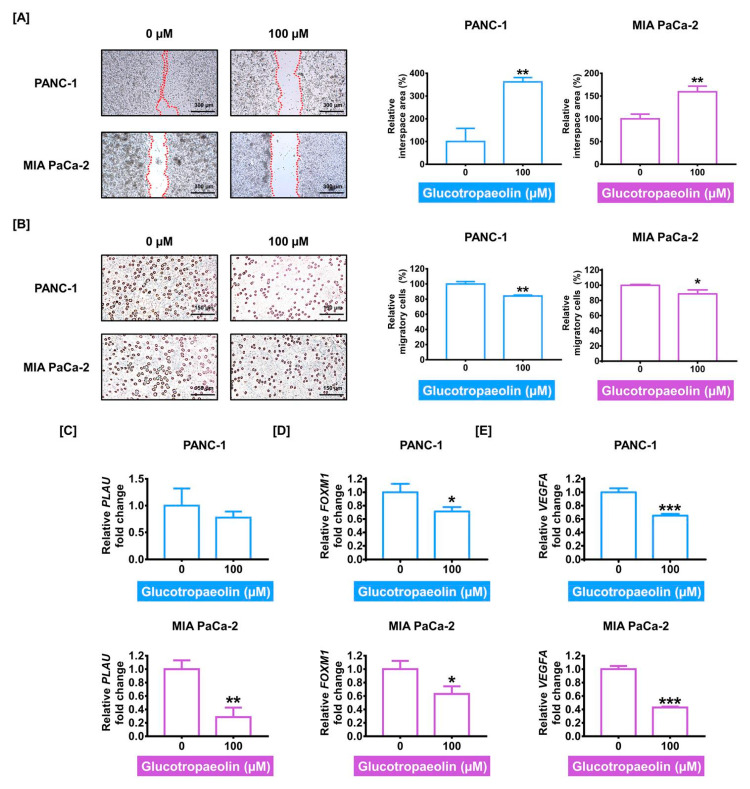
Attenuated invasiveness of pancreatic ductal adenocarcinoma (PDAC) cells by glucotropaeolin (GT) treatment. (**A**) Using ibidi 35 mm dishes, the cell invasion test was performed with GT treatment for 24 h in PDAC cells. Relative interspace area was measured as the indicator for cell invasiveness. Scale bar: 300 µm. (**B**) Cell migration assay was performed to measure migratory levels of PDAC cells following GT treatment for 24 h. Scale bar: 150 µm. (**C**–**E**) Invasive gene expressions of (**C**) PLAU, (**D**) FOXM1, and (**E**) VEGFA, which were induced by GT, were evaluated by qPCR. These gene expressions were quantified with GAPDH expression. Triplicated experiments were performed. The levels of statistical significance between control and GT-treated groups are indicated by asterisks, as confirmed by *t*-test analysis (* *p* < 0.05, ** *p* < 0.01, and *** *p* < 0.001).

Next, we evaluated whether GT could suppress PDAC cell invasion and migration. First, in the invasion assay, the interspace area, which is inversely proportional to the cell invasion ability, was considerably increased by GT treatment, by up to 362.2% (PANC-1) and 159.5% (MIA PaCa-2) (both *p* < 0.01), compared to the control group (Figure 5A). Moreover, in the migration assay, the migratory cells were significantly decreased by 100 µM of GT (Figure 5B). The reduced effects of GT on cell migration were 15.9% (PANC-1) and 11.4% (MIA PaCa-2) (*p* < 0.01 and *p* < 0.05, respectively). Subsequently, we further confirmed the transcriptional levels of migratory genes by performing qPCR. The mRNA expression of plasminogen activator urokinase (PLAU) in PANC-1 was 0.78-fold in response to GT treatment compared to control, which was not significant. However, its mRNA expression in MIA PaCa-2 was significantly reduced to 0.29-fold (*p* < 0.01) compared to the control (Figure 5C). Additionally, mRNA levels of FOXM1 were also decreased to 0.71-fold (PANC-1) and 0.63-fold (MIA PaCa-2) (both *p* < 0.05), in response to GT (Figure 5D). Moreover, vascular endothelial growth factor A (VEGFA) mRNA expression was also reduced to 0.65-fold (PANC-1) and 0.43-fold (MIA PaCa-2) by GT (both *p* < 0.001) (Figure 5E). Therefore, our observations suggested that GT undermined the migratory ability of PDAC cells. 

### 3.6. Efficacy of GT Is Strengthened by FOXM1 Silencing via Calcium Dysregulation in PDAC Cells

**Figure 6:**
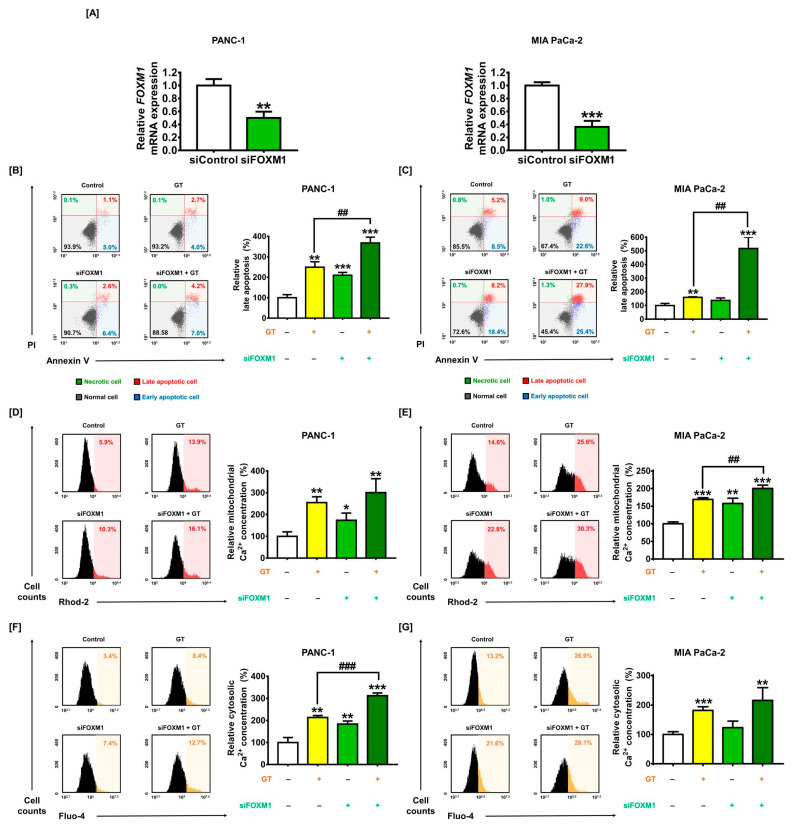
*FOXM1* knockdown contributes to glucotropaeolin (GT)-caused the induction of apoptosis and calcium dysregulation. (**A**) Knockdown of *FOXM1* after 50 nM siFOXM1 transfection for 24 h in pancreatic ductal adenocarcinoma (PDAC) cells was measured by qPCR. (**B**,**C**) The apoptotic cell rate of PDAC cells was measured by Annexin V/PI staining with flow cytometry, following siFOXM1 (50 nM) transfection for 24 h and GT treatment for 48 h. (**D**,**E**) The alteration of mitochondrial calcium concentration in PDAC cells was evaluated using Rhod-2 after siFOXM1 (50 nM) transfection for 24h and GT treatment for 48 h. (**F**,**G**) The alterations to cytosolic calcium concentrations in PDAC cells were evaluated by Fluo-4 after siFOXM1 (50 nM) transfection for 24h and GT treatment for 48 h. Triplicated experiments were performed. The levels of statistical significance between the control and GT-treated group are indicated by asterisks (* *p* < 0.05, ** *p* < 0.01, *** *p* < 0.001), and between the GT-treatment group and the GT-treated-simultaneously-siFOXM1-transfected group by crosshatches (## *p* < 0.01 and ### *p* < 0.001), as confirmed by *t*-test analysis or one-way analysis of variance (ANOVA), followed by Tukey’s post hoc test.

Evidence has shown that FOXM1 is deeply involved in tumor progression, promotion, and the migration of PDAC [29,30]. To evaluate the contribution of FOXM1 as a proper anticancer target, we examined intracellular changes in FOXM1-attenuated PDAC cells by transfection with siRNA, namely siFOXM1. At an siFOXM1 concentration of 50 nM, the mRNA expression of FOXM1 was effectively reduced to 50.0% (PANC-1) and 36.2% (MIA PaCa-2) (*p* < 0.01 and *p* < 0.001, respectively), compared to the control siRNA (siControl) (Figure 6A). Then, we examined the functional role of FOXM1 related to the GT-induced anticancer activity in PDAC cells. First, the Annexin V/PI assay indicated that FOXM1 knockdown increased the late apoptosis of PANC cells compared to the control (from 1.1% to 2.6%; *p* < 0.001) and elevated the anticancer effect of GT from 2.7% to 4.2% (*p* < 0.01) (Figure 6B). In MIA PaCa-2, although siFOXM1 (50 nM) transfection cannot significantly influence the alteration of the late apoptosis rate compared to the control (from 6.2% to 8.2%), the combination of GT with siFOXM1 considerably increased the number of late apoptotic cells from 9.0% to 27.9% (*p* < 0.01) (Figure 6C). In PDAC cells, transfection with siFOXM1 (50 nM) enhanced mitochondrial calcium levels compared to the control, from 5.9% to 10.3% and from 14.6% to 22.8% (*p* < 0.05 and *p* < 0.01, respectively) (Figure 6D,E). Compared to GT-treated cells, the combination of GT and siFOXM1 significantly increased mitochondrial calcium concentrations from 25.6% to 30.3% in MIA PaCa-2 (*p* < 0.01). Moreover, in PANC-1, siFOXM1 transfection increased cytosolic calcium levels compared to the control from 3.4% to 7.4% (*p* < 0.01). However, FOXM1 knockdown did not change the cytosolic calcium level in MIA PaCa-2. FOXM1 knockdown also increased cytosolic calcium levels of GT-treated PANC-1 cells from 8.4% to 12.7% (*p* < 0.001) (Figure 6F,G). Our findings imply that the knockdown of FOXM1 in PDAC strengthens the antitumor activity of GT by stimulating apoptosis and disrupting calcium homeostasis.

### 3.7. Migratory Ability Is Attenuated by FOXM1 Silencing in PDAC Cells

**Figure 7:**
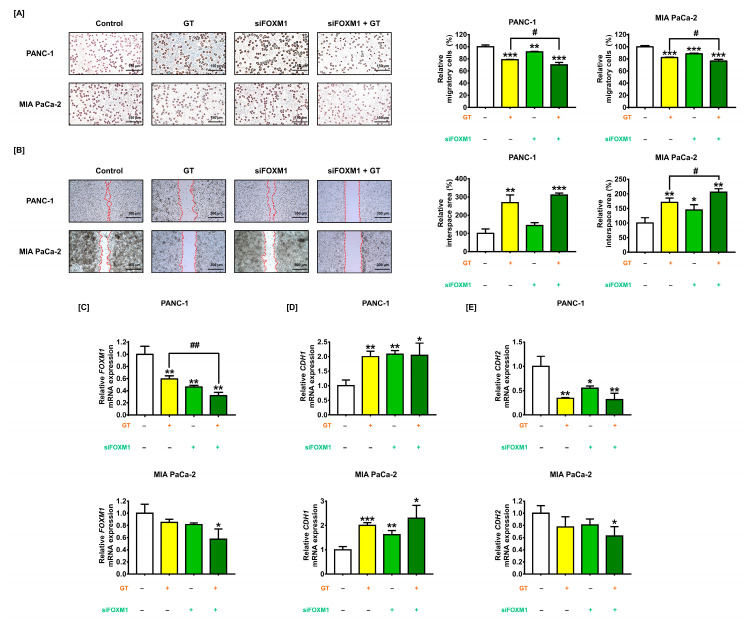
FOXM 1 knockdown further weakens glucotropaeolin (GT)-induced inhibition of cell migration and invasion in pancreatic ductal adenocarcinoma (PDAC) cells. (**A**) With PDAC cells transfected with siFOXM1 (50 nM), cell migration analysis was conducted to measure migratory levels following GT treatment. Scale bar: 150 µm. (**B**) With PDAC cells transfected with siFOXM1 (50 nM), cell invasion analysis was conducted in response to GT treatment, using an ibidi 35 mm dish. Relative interspace area was measured as the indicator for cell invasiveness. Scale bar: 300 µm. (**C**–**E**) After the silencing of *FOXM1* with 50 nM siFOXM1 transfection for 24 h in PDAC cells, the mRNA expressions of (**C**) *FOXM1*, (**D**) *CDH1*, and (**E**) *CDH2* were measured by qPCR. Triplicated experiments were performed. The levels of statistical significance between the control and the GT-treated group are indicated by asterisks (* *p* < 0.05, ** *p* < 0.01, *** *p* < 0.001), and between the GT-treatment group and GT-treated-simultaneously-siFOXM1-transfected group by crosshatches (# *p* < 0.05 and ## *p* < 0.01), as confirmed by one-way analysis of variance (ANOVA), followed by Tukey’s post hoc test.

To investigate the contribution of FOXM1 on the suppressive effects of GT on PDAC cell invasiveness, we conducted a cell migration and invasion assay with siFOXM1-transfected PDAC cells. The results of the migration assay indicated that compared to the control group, inhibitory effects of FOXM1 knockdown were 8.6% (PANC-1) and 11.7% (MIA PaCa-2) (*p* < 0.01 and *p* < 0.001 respectively) (Figure 7A). Compared to GT-treated PDAC cells, the transfection of siFOXM1 also enhanced the inhibition ability of GT to cell migration from 21.3% to 30.0% (PANC-1) and from 17.7% to 23.4% (MIA PaCa-2) (both *p* < 0.05). Additionally, in the ibidi invasion assay, compared to the control group, FOXM1 knockdown did not affect the invasiveness of PANC-1; however, it increased that of MIA PaCa-2 by up to 144.8% (*p* < 0.05). Moreover, siFOXM1 transfection in MIA PaCa-2 strengthened the inhibitory effect of GT on cell invasion from 171.1% to 205.7% (*p* < 0.05), compared to GT-treated MIA PaCa-2 cells (Figure 7B). Subsequently, we further confirmed the changes in the mRNA expression of invasive genes induced by FOXM1 knockdown and GT treatment. FOXM1 knockdown in PANC-1 and MIA PaCa-2 reduced FOXM1 transcriptional levels from 60% to 32% (*p* < 0.01) and from 85% to 57%, respectively, compared to GT-treated PDAC cells (Figure 7C). The combination of GT with siFOXM1 transfection significantly increased the mRNA level of cadherin 1(CDH1) in both PDAC cells (both *p* < 0.05) (Figure 7D). Furthermore, the transcriptional level of cadherin 2 (CDH2) was significantly reduced by the combination of GT with siFOXM1 transfection in both PDAC cells (Figure 7E). Collectively, these observations suggest that FOXM1 downregulation in PDAC cells further attenuates the migratory ability of PDAC cells, which is inhibited by GT.

## 4. Discussion

**Figure 8:**
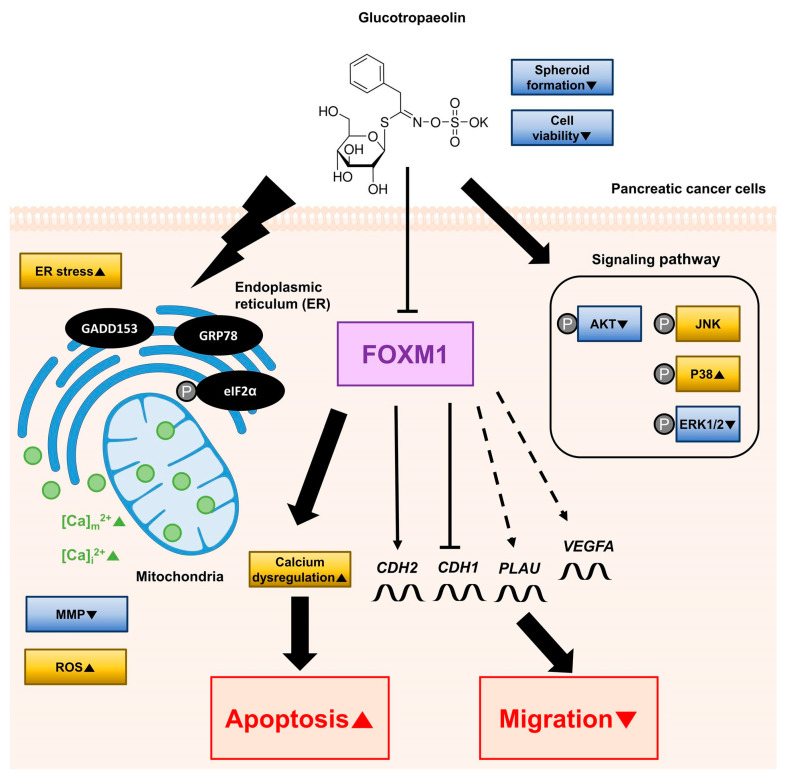
A schematic diagram depicting the anticancer effect of glucotropaeolin on pancreatic ductal adenocarcinoma cells. Dashed lines represent interactions that are not direct.

In this study, our observations indicated that GT exhibits anticancer activity in PDAC cells. GT suppressed cell proliferation and spheroid formation in PDAC cells. Additionally, GT disrupted mitochondrial physiological conditions in terms of MMP reduction and ROS accumulation. We also verified that GT induces apoptosis and hampers calcium homeostasis. Moreover, GT attenuated cell migration by regulating EMT-associated genes. Furthermore, GT stimulated ER stress-related proteins and modulated AKT and MAPK signaling pathways participating in cell progression and proliferation in PDAC cells. We revealed that the inhibitory effect of GT on cell migration and the induction of apoptosis by calcium dysregulation were subjected to the FOXM1 regulation in PDAC cells. Altogether, we clarified the intracellular mode of action underlying the antitumor activity of GT in PDAC cells (Figure 8).

GT is a glucosinolate that can be extracted from the *Brassicaceae* family such as cruciferous vegetables [8]. Numerous previous reports have suggested that glucosinolates and their breakdown products exhibit anticancer and cancer-protective activity through the induction of apoptosis in various types of cancer. Benzyl isothiocyanate (BITC), a breakdown product from GT, activates the apoptosis signaling pathway involved in ROS-stimulated mitochondrial dysfunction in gastric adenocarcinoma cells [31]. Moreover, BITC promotes apoptosis and suppresses the invasiveness of hepatocellular carcinoma [32]. Glucosinolates extracted from *Brassica oleracea var. acephala* induce apoptosis by increasing the protein expression of BAX and cytochrome c in human prostate cancer [33]. We first reveal the anticancer effect of GT in PDAC cells. Consistent with the previous reports, our results clearly indicated that GT suppressed cell proliferation and triggered the induction of apoptosis in PDAC cells. Spheroid formation in a 3D environment was inhibited by GT treatment in PDAC cells, suggesting that GT has the potency to regulate tumor formation in PDAC cells. Moreover, the capability of GT to promote apoptosis in PDAC cells is further confirmed by the elevated cytochrome c and BAX and reduced Bcl-xL in PDAC cells in response to GT treatment. In apoptosis through mitochondria, cytochrome c released from mitochondria can stimulate caspase-9 and caspase-3, which have crucial roles in intrinsic and extrinsic pathways, respectively [34,35]. Proapoptotic protein BAX activation is also indispensable to mitochondria-dependent apoptosis [36]. Since overexpressed antiapoptotic Bcl-xL protein in PDAC facilitated cancer cells to escape the apoptotic process, Bcl-xL inhibitors have been a strong candidate for a new treatment option for PDAC [37]. Although detailed descriptions regarding the interaction between BAX and Bcl-xL during the apoptosis process induced by GT in PDAC cells are needed, it could be concluded that GT activates intrinsic apoptosis in PDAC cells via the upregulation of BAX and inhibition of Bcl-xL.

Calcium signaling is an important modulator of apoptosis, tumor metastasis, and proliferation in cancer [38,39]. Calcium overload in mitochondria can trigger the stimulation of proapoptotic proteins and reduce MMP to disrupt mitochondrial function [40,41]. Moreover, cytosolic free calcium ions upregulate proapoptotic factors such as BAD and suppress antiapoptotic protein (BCL-2), which causes the apoptosis process [42]. Additionally, the interplay between calcium signaling and ROS is closely related to the process of apoptosis in cancer [43]. To explain the comprehensive calcium regulation by GT in PDAC cells, in our study, we used BAPTA/AM and 2-APB, which act as calcium chelators. BAPTA/AM can prevent the deregulation of calcium ions and protect cells from calcium influx as a calcium chelator [44], and 2-APB, which is usually used for a calcium probe, exerts an antagonistic effect on calcium entry [45]. GT elevated the calcium concentrations of mitochondria and cytosol, which is further confirmed by the co-treatment of GT with BAPTA/AM or 2-APB, suggesting that GT promotes calcium overload in mitochondria and cytosol. Additionally, we confirmed the ROS accumulation and MMP reduction induced by GT in PDAC cells, implying that ROS production and the loss of MMP resulted from the process of apoptosis in PDAC cells. Therefore, our findings further suggest that GT promotes the apoptosis of PDAC cells via calcium dysregulation in mitochondria.

Cancer cells extend from the original tumor’s peripheral organs, followed by cancer cell migration to create new tumors during metastasis. Some cancer cells can be under EMT, thus losing epithelial properties and acquiring mesenchymal characteristics such as motility, invasiveness, and migration [46]. EMT even contributes to drug resistance and apoptosis evasion in PDAC [47]. Therefore, evidence has suggested that EMT-related genes may be targets to treat PDAC. For instance, plasminogen activator urokinase (PLAU), which is overexpressed in PDAC tissue and can be used for the worse prognostic marker in a clinical cohort, may be a therapeutic target, by inhibiting PLAU expression to attenuate the proliferation and metastasis of PDAC [48,49]. Additionally, approximately 93% of patients with PDAC exhibit vascular endothelial growth factor A (VEGFA) expression, which is also a marker for a worse prognosis and predictor for liver metastasis in PDAC [50]. VEGF-targeted therapy can suppress the migration, invasion, and proliferation of PDAC [51]. Moreover, the downregulation of *FOXM1*, which has an important role in the EMT of PDAC, can be a therapeutic strategy for the treatment of PDAC [52]. Consistent with the previous studies, our qPCR results confirmed that GT treatment reduced the mRNA level of representative migratory genes, including *PLAU*, *VEGFA*, and *FOXM1*. Additionally, our results suggested that GT can hamper the migration of PDAC cells. Therefore, our findings further suggested that GT attenuated cell invasion and migration via the regulation of EMT-related genes in PDAC cells. It can be speculated that attenuation of *PLAU* and *VEGFA* induced by GT in PDAC is mediated by FOXM1 regulation. Further studies are needed on whether *PLAU* and *VEGFA* can be regulated by FOXM1 in PDAC cells.

The permanent activation of KRAS is an essential mutation for the initiation, progression, and maintenance of tumorigenesis in PDAC [53]. Many efforts have tried to develop the drug against PDAC aiming to direct the inhibition of KRAS, but the high risk of relapse is followed by treatment with *KRAS* inhibitors [54]. The recent strategies for the inactivation of KRAS have focused on the indirect inhibition of KRAS by blocking downstream effectors, RAF-MEK-ERK and PI3K-AKT-mTOR signaling pathways [55]. Trametinib and selumetinib (oral MEK1/2 inhibitors) and a combination of AKT inhibitors with mTOR inhibitors have been developed [56]. We found that GT treatment suppressed the phosphorylated AKT and ERK1/2 MAPK in PDAC cells. These observations are consistent with previous studies. Moreover, we confirmed the levels of ER stress proteins (GRP78, GADD153, and phosphorylated eIF2α) induced by GT in PDAC cells. Several pieces of evidence have suggested that anticancer properties can be exerted by activating the ER stress pathway in PDAC cells, for example, the activation of PERK-eIF2α-CHOP signaling triggered apoptosis in PDAC [57]. Moreover, enhanced ER stress signaling drives the induction of apoptosis through ROS-mediated Ca^2+^ signaling in PDAC cells [58]. Collectively, our results imply that GT exhibits an anticancer effect through the regulation of MAPK, AKT, and ER stress signaling, which is involved in PDAC cell survival.

FOXM1 is a fundamental transcriptional factor that is involved in the tumor invasion, metastasis, and progression of various cancers [59]. Evidence suggests that FOXM1 is a key regulator of EMT activation in PDAC [60]. In addition, Xin Li and colleagues demonstrated that FOXM1 in PDAC cells modulated the expression of E-cadherin and N-cadherin, encoded by *CDH1* and *CDH2*, respectively [61]. Moreover, the knockdown of *FOXM1* in the human cancer cell can be susceptible to apoptosis, including PDAC cells [62]. Furthermore, some studies reported that the knockdown of *FOXM1* in PDAC cells enhanced gemcitabine sensitivity [23,63], thus, suggesting that GT may exert anticancer properties as a chemotherapeutic adjuvant in combination with gemcitabine in PDAC. Our observations indicate that the induction of apoptosis of GT is accelerated by *FOXM1* silencing via the calcium dysregulation of PDAC cells. Furthermore, the knockdown of *FOXM1* further weakened the invasiveness of PDAC cells with GT treatment. Therefore, our findings imply that FOXM1 has an important role in the anticancer activity of GT in terms of the induction of apoptosis and migration suppression. The limitations of the present study are firstly that PDAC cells were incubated in serum-free medium for 24 h before the GT treatment. This condition is somewhat different from clinical environment of cancer cells. Secondly, the toxicity of GT in noncancer cell lines was not confirmed. Further studies are needed to establish whether GT exhibits cytotoxicity in noncancer cell lines.

## 5. Conclusions

This study first verified the anticancer properties of GT in pancreatic cancer cells. GT hampered PDAC cell progression and migration. Additionally, GT increased ROS generation, the rate of apoptotic cells, MMP disruption, and calcium dysregulation in PDAC cells. Moreover, the inhibition of the FOXM1 expression elevated the potentiation of apoptosis by GT and the inhibitory effect of GT on cell migration. Altogether, our study demonstrates that GT can be a potential therapeutic drug against PDAC. Since our study was limited to experiments with cancer cell lines and shows the limitations presented in the last paragraph of the Discussion, further in vitro and in vivo studies are required to confirm the usefulness of glucotropaeolin in the treatment of pancreatic ductal adenocarcinoma.

## Figures and Tables

**Table 1:** The detailed information about antibodies we used.

Antibody	Supplier	Dilution	Catalog Number
Bcl-xL	Proteintech	1:1000	10783-1-AP
BAX	Proteintech	1:2000	50599-2-Ig
Cytochrome c	Proteintech	1:1000	66264-1-Ig
GADD153	Proteintech	1:1000	15204-1-AP
GRP78	Proteintech	1:1000	11587-1-AP
p-eIF2α (Ser51)	Cell Signaling Technology	1:1000	3398
eIF2α	Cell Signaling Technology	1:1000	5324
p-ERK1/2 (Thr202/Tyr204)	Cell Signaling Technology	1:1000	9101
ERK1/2	Cell Signaling Technology	1:1000	4695
p-JNK (Thr183/Tyr185)	Cell Signaling Technology	1:1000	4668
JNK	Cell Signaling Technology	1:1000	9252
p-P38 (Thr180/Tyr182)	Cell Signaling Technology	1:1000	4511
P38	Cell Signaling Technology	1:1000	9212
p-AKT (Ser473)	Cell Signaling Technology	1:1000	4060
AKT	Cell Signaling Technology	1:1000	9272
β-actin	Santa Cruz Biotechnology	1:1000	sc-47778

**Table 2:** The primers we used in qPCR.

Gene	Size (bp)	GenBank Accession No.	Primer Sequence (5′→3′)
*forkhead box protein M1 (FOXM1)*	104	NM_001243088.2	F: AGTCACACCCTAGCCACTGC
			R: ACCATTGCCTTTGTTGTTCC
*plasminogen activator, urokinase (PLAU)*	139	NM_002658.6	F: TGTGAGATCACTGGCTTTGG
			R: TTTTGGTGGTGACTTCAGAG
*vascular endothelial growth factor A (VEGFA)*	109	NM_001025366.3	F: CTGCTCTACCTCCACCATGC
			R: AGCTGCGCTGATAGACATCC
*cadherin 1 (CDH1)*	112	NM_001317184.2	F: CGTAGCAGTGACGAATGTGG
			R: TTCAGGAGGCACAAAGATGG
*cadherin 2 (CDH2)*	103	NM_001308176.2	F: AGGTTTGCCAGTGTGACTCC
			R: ATGATGCAGAGCAGGATGG
*glyceraldehyde-3-phosphate dehydrogenase (GAPDH)*	149	NM_001256799.3	F: GGCTCTCCAGAACATCATCC
			R: TTTCTAGACGGCAGGTCAGG

## Data Availability

Data is contained within the article.
